# CFTR Deficiency Disrupts Bladder Function Through Ion Imbalance and Inflammatory–Apoptotic Signaling

**DOI:** 10.3390/ijms27146448

**Published:** 2026-07-20

**Authors:** Kuo-Chiang Chen, Huei-Jiun Tzeng, Meng-Lin Chang, Chellappan Praveen Rajneesh, Han-Sun Chiang, Wen-Chun Hsu, Hung-Chune Maa, Yi-No Wu

**Affiliations:** 1 School of Medicine, College of Medicine, Fu Jen Catholic University, New Taipei City 242062, Taiwan; kkhk88@gmail.com (K.-C.C.); oro.tidyscoundrel@gmail.com (M.-L.C.); praveenrajneesh@gmail.com (C.P.R.); 2 Department of Urology, Cathay General Hospital, Taipei City 10630, Taiwan; 3 Program in Pharmaceutical Biotechnology, College of Medicine, Fu Jen Catholic University, New Taipei City 242, Taiwan; cherry910502@gmail.com; 4 Department of Urology, Fu Jen Catholic University Hospital, Fu Jen Catholic University, New Taipei City 243089, Taiwan; 053824@mail.fju.edu.tw; 5 Graduate Institute of Nutrition and Food Sciences, Fu Jen Catholic University, New Taipei City 242062, Taiwan; 6 Department of Clinical Pathology, Cathay General Hospital, Taipei City 10630, Taiwan; bsc@cgh.org.tw; 7 Department of Pathology, Cardinal Tien Hospital, New Taipei City 231403, Taiwan

**Keywords:** CFTR deficiency, bladder dysfunction, cystometry, detrusor contractility, ion imbalance, urothelium, inflammation, apoptosis, COX-2, caspase-9

## Abstract

The cystic fibrosis transmembrane conductance regulator (CFTR) is a key determinant of epithelial ion transport; however, its role in lower urinary tract physiology remains unclear. This study investigated whether CFTR deficiency disrupts bladder function by altering ionic homeostasis and downstream cellular signaling. Bladder function was evaluated in 12-month-old CFTR knockout (Cftr^−^/^−^) and wild-type mice (*n* = 8/group) using in vivo cystometry and ex vivo detrusor contractility assays, in addition to histological, immunofluorescence, electrolyte, and Western blot analyses. Cftr^−^/^−^ mice exhibited unstable cystometric profiles with irregular voiding cycles and significantly increased peak voiding pressure, indicating impaired bladder coordination. In contrast, depolarization-induced detrusor contractility was markedly reduced (0.3855 g vs. 1.908 g in wild type), despite preserved bladder morphology and unchanged α-SMA expression. CFTR deficiency was further associated with selective electrolyte imbalance (decreased Na^+^ and Cl^−^, increased K^+^). At the molecular level, reduced cytokeratin 20 expression (*p* < 0.01) suggested urothelial impairment, whereas increased COX-2 (*p* < 0.05) and caspase-9 (*p* < 0.01) indicated activation of inflammatory and apoptotic pathways. Collectively, these findings demonstrate that CFTR deficiency disrupts bladder functional homeostasis through integrated effects on ion balance, detrusor excitability and cellular signaling.

## 1. Introduction

The cystic fibrosis transmembrane conductance regulator (CFTR) is a chloride channel that plays a critical role in epithelial ion transport and fluid homeostasis [1]. Mutations in CFTR cause cystic fibrosis, a multisystem disorder that primarily affects the respiratory and gastrointestinal systems [2]. In addition to its well-established role in epithelial tissues, CFTR is increasingly recognized as an important regulator of ion transport and cellular homeostasis in other organ systems [3]. Recent studies have suggested that CFTR activity may influence smooth muscle physiology and cellular ionic balance across multiple tissues, indicating that its functional role extends beyond classical epithelial functions [4,5,6,7].

**Figure 1:**
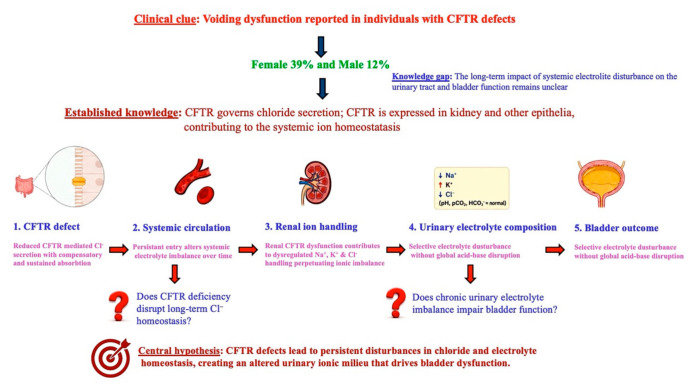
Proposed conceptual framework linking systemic CFTR deficiency to bladder dysfunction through chronic electrolyte imbalances. Clinical evidence indicates an increased prevalence of voiding dysfunction in individuals with CFTR defects; however, the contribution of chronic systemic electrolyte disturbances remains unclear. CFTR-mediated chloride transport is essential for maintaining systemic ion balance. The proposed model suggests that CFTR deficiency disrupts chloride transport, producing a persistent systemic electrolyte imbalance that is further amplified by impaired renal ion handling. This results in selective disturbances in Na^+^, K^+^, and Cl^−^ homeostasis, while preserving the overall acid–base balance. The resulting chronic alteration of the urinary ionic milieu is hypothesized to impose sustained stress on the bladder, ultimately contributing to its dysfunction. The figure also highlights two key mechanistic questions addressed in this study: whether CFTR deficiency disrupts long-term Cl^−^ homeostasis, and whether chronic urinary electrolyte imbalance impairs bladder function. Cl^−^, chloride; Na^+^, sodium; K^+^, potassium; pH, potential of hydrogen; pCO_2_, partial pressure of carbon dioxide; HCO_3_**^−^**, bicarbonate.

The urinary bladder is a highly dynamic organ, and its function depends on the coordinated interactions between detrusor smooth muscle contractility, neural regulation [8], and ionic balance within the bladder tissues [9]. Proper regulation of ionic flux across cellular membranes is essential for maintaining detrusor excitability and contractile responses during bladder filling and voiding cycles. Therefore, disruption of ion transport mechanisms can alter bladder pressure dynamics and smooth muscle responsiveness, potentially leading to urinary dysfunction [10]. Animal models have provided valuable insights into the systemic physiological consequences of CFTR dysfunction, beyond the classical manifestations of cystic fibrosis. In particular, few studies have examined whether CFTR dysfunction influences detrusor muscle contractility or bladder urodynamic parameters [11,12]. Loss of CFTR-mediated Cl^−^ transport may secondarily alter Na^+^ handling via ENaC dysregulation, with indirect effects on K^+^ homeostasis. Detrusor smooth muscle contraction depends on membrane depolarization and subsequent calcium (Ca^2+^) influx through voltage-gated calcium channels; thus, such ionic disturbances may influence membrane potential and excitation–contraction coupling. A schematic representation of these mechanisms is presented in Figure 1.

Figure 1 summarizes the conceptual framework of the present study. Clinical evidence indicates an increased prevalence of voiding dysfunction in individuals with CFTR defects, although the long-term effects of systemic electrolyte imbalances on bladder function remain unclear. Given the established role of CFTR in chloride transport and systemic ion homeostasis, we hypothesized that CFTR deficiency produces persistent disturbances in chloride and electrolyte homeostasis through impaired renal ion handling, leading to selective electrolyte imbalance without global acid–base disruption. Chronic alterations in the urinary ionic milieu are proposed to promote bladder dysfunction. The figure also highlights the two key mechanistic questions investigated in this study: whether CFTR deficiency disrupts long-term Cl^−^ homeostasis and whether chronic urinary electrolyte imbalance contributes to bladder dysfunction.

Recent experimental evidence has highlighted the potential role of CFTR-mediated ion transport in the lower urinary tract [13]. CFTR is expressed in both the urothelium and smooth muscle layers of the bladder, where it may contribute to chloride and bicarbonate transport, epithelial barrier regulation, and smooth muscle excitability [14]. Disruption of CFTR-mediated ion transport may secondarily alter the ionic microenvironment surrounding the detrusor smooth muscle, thereby influencing bladder contractility. Such changes in the urinary bladder can affect detrusor responsiveness and bladder pressure regulation, leading to functional disturbances even in the absence of overt structural abnormalities. However, the precise contribution of CFTR to bladder urodynamics and smooth muscle physiology remains to be elucidated. Therefore, this study investigated whether CFTR deficiency disrupts bladder function by altering ionic homeostasis and downstream cellular signaling in the absence of structural remodeling.

## 2. Results

### 2.1. Altered Cystometric Profiles in CFTR-Deficient Mice

**Figure 2:**
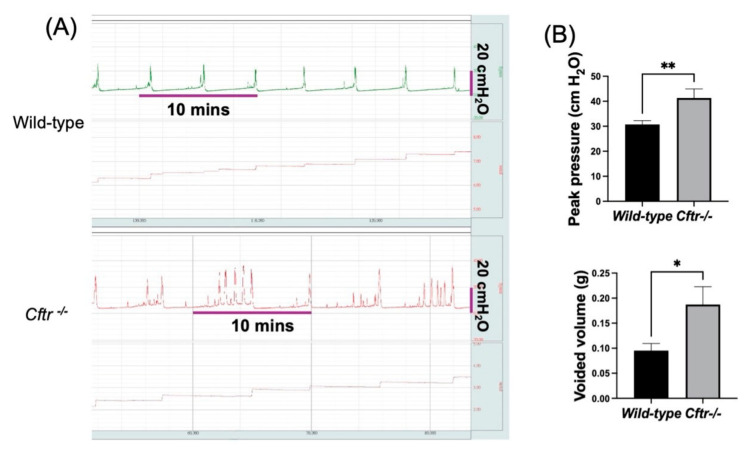
Cystometric analysis demonstrated bladder dysfunction in Cftr^−^/^−^ mice. (**A**) Representative cystometrogram (CMG) traces recorded over a 10-min period in wild-type and Cftr^−^/^−^ mice. Cftr^−^/^−^ mice exhibited frequent pressure fluctuations and irregular voiding contractions compared to wild-type mice. (**B**) Quantitative analysis showing significantly increased peak intravesical pressure and voided volume in Cftr^−^/^−^ mice. These findings indicate enhanced detrusor contractile activity and altered bladder emptying in the absence of CFTR expression. Data are presented as mean ± SEM. * *p* < 0.05, ** *p* < 0.01 vs. wild-type.

Cystometric analysis was performed in 12-month-old wild-type and Cftr^−^/^−^ mice to evaluate their bladder function (Figure 2).

Representative cystometrograms from wild-type mice showed regular filling phases followed by coordinated voiding contractions with stable intercontraction intervals, reflecting normal micturition. In contrast, Cftr^−^/^−^ mice exhibited an altered pattern characterized by irregular contraction and frequent pressure fluctuations. Voiding contractions were less coordinated and were often preceded by multiple smaller pressure spikes. Quantitative analysis revealed that the peak voiding pressure was significantly higher in Cftr^−^/^−^ mice than in wild-type (WT) controls. These results indicate impaired bladder pressure regulation and altered micturition dynamics in the absence of CFTR.

### 2.2. Histological Assessment of Bladder Morphology

**Figure 3:**
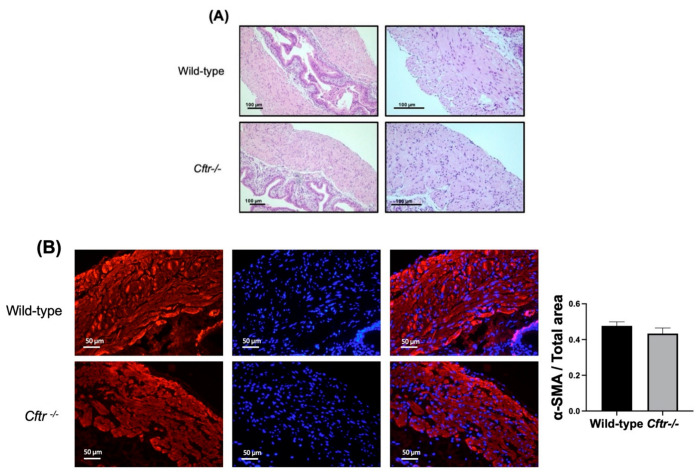
Histological assessment of bladder tissue architecture in wild-type and Cftr^−^/^−^ mice. (**A**) Representative hematoxylin and eosin (H&E) staining of bladder sections from wild-type and Cftr^−^/^−^ mice, showing the urothelial layer, lamina propria, and detrusor muscle. Overall, the tissue organization was preserved in both groups. (**B**) Higher magnification images highlighting the detrusor smooth muscle structure. No obvious structural disruption was observed in the wild-type and Cftr^−^/^−^ mice. Scale bars are indicated in the images.

To determine whether functional alterations were associated with structural changes, histological analysis was performed on the bladder tissues from both groups (Figure 3). Hematoxylin and eosin staining revealed that the overall architecture of the bladder wall, including the urothelium, lamina propria, and detrusor muscle layers, was preserved in both wild-type and Cftr^−^/^−^ mice. Immunofluorescence staining for α-smooth muscle actin (α-SMA) demonstrated a similar distribution of smooth muscle fibers in both the groups.

Quantitative analysis of the α-SMA-positive area revealed no significant difference between the CFTR knockout and wild-type mice. These findings indicate that CFTR deficiency does not induce overt structural remodeling of the bladder wall.

### 2.3. Detrusor Contractility Is Reduced in CFTR Knockout Mice

**Figure 4:**
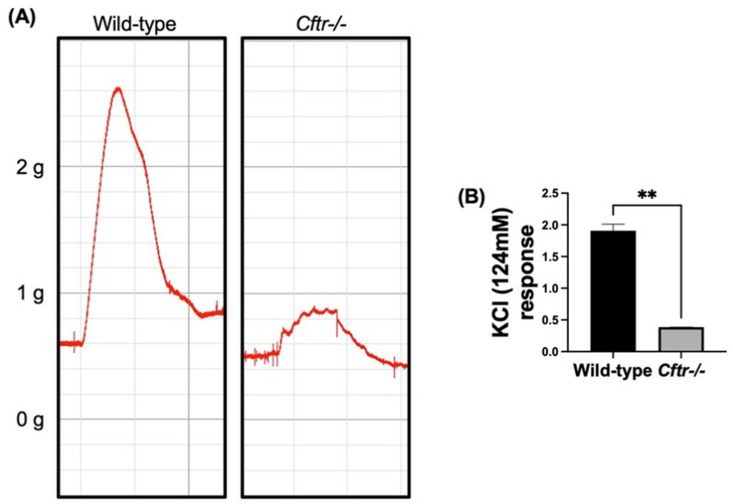
Altered detrusor contractility in bladder tissues of Cftr^−^/^−^ mice. (**A**) Representative contractile tension recordings from isolated bladder strips stimulated with a high potassium (K^+^) solution. (**B**) Quantitative analysis showing significantly increased contractile responses in Cftr^−^/^−^ bladder tissues compared with wild-type controls, indicating enhanced detrusor smooth muscle excitability. Data are presented as mean ± SEM. ** *p* < 0.05 vs. wild-type.

Histological analysis revealed no structural abnormalities in the bladder tissue. To evaluate the intrinsic detrusor function, organ bath experiments were performed using a high-potassium Krebs solution (124 mM KCl) to induce depolarization-dependent contractions (Figure 4A). Bladder strips from wild-type mice exhibited robust contractile responses, with a rapid increase in tension, followed by gradual relaxation. The average peak tension was 1.908 g (Figure 4B).

In contrast, bladder strips from Cftr^−^/^−^ mice showed markedly attenuated contractile responses, with reduced amplitude and a blunted peak. The average contractile force was significantly lower (0.3855 g vs. 1.908 g, unpaired *t*-test). These findings demonstrate impaired depolarization-induced contractility in the CFTR-deficient detrusor smooth muscle. 

### 2.4. Systemic Ion Profile in Cftr^−^/^−^ Mice

**Figure 5:**
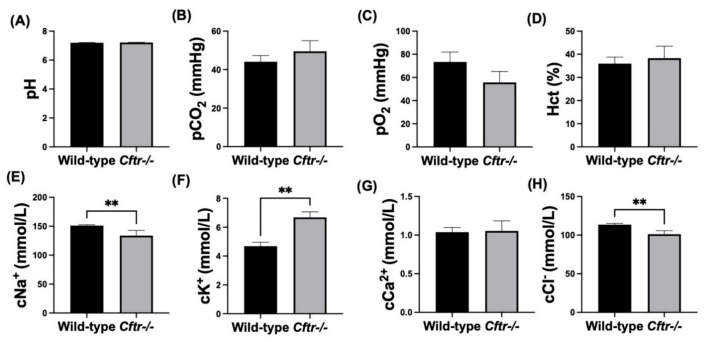
Systemic blood gas and electrolyte parameters in wild-type (CFTR^+^/^+^) and Cftr^−^/^−^ mice. Whole blood was collected from adult mice (12 months old) under anesthesia immediately after cystometric recording. Blood gas and electrolyte analyses were performed using a point-of-care analyzer. (**A**–**D**) Blood gas parameters: pH, partial pressure of carbon dioxide (pCO_2_), partial pressure of oxygen (pO_2_), and hematocrit (Hct) showed no statistically significant differences between groups, although a consistent trend toward reduced pO_2_ was observed in Cftr^−^/^−^ mice. (**E**–**H**) Electrolyte profile: Sodium (Na^+^) and chloride (Cl^−^) concentrations were significantly reduced in Cftr^−^/^−^ mice, whereas potassium (K^+^) was significantly elevated. The calcium (Ca^2+^) levels did not differ significantly between the groups. ** *p* < 0.05 vs. wild-type.

To assess whether systemic ionic alterations were present, whole blood samples were analyzed for blood gas and electrolyte parameters (Figure 5A–H). Whole-blood gas parameters were evaluated to determine whether CFTR deficiency alters the systemic acid–base balance or oxygenation status (Figure 5A–D). Although pO_2_ values showed a consistent trend toward reduction in Cftr^−^/^−^ mice, this difference did not reach statistical significance (*p* > 0.05). Global respiratory and acid–base homeostasis remained largely preserved, as evidenced by comparable pH, pCO_2_, and hematocrit values between the groups.

In contrast, the analysis of circulating electrolytes revealed significant alterations in the levels of specific ions (Figure 5E–H). The decrease in Cl^−^ levels is consistent with the direct loss of CFTR-mediated anion transport at the epithelial apical membrane. The reduction in Na^+^ is likely a secondary consequence of CFTR-dependent regulation of ENaC activity, whereby the absence of CFTR leads to dysregulated epithelial Na^+^ absorption and subsequent depletion of circulating Na^+^. Potassium (K^+^) levels were significantly elevated, likely reflecting an indirect compensatory response to disrupted Na^+^/Cl^−^ homeostasis, rather than a direct effect of CFTR. The calcium (Ca^2+^) concentrations remained unchanged, consistent with the absence of direct CFTR-mediated Ca^2+^ transport regulation.

### 2.5. Molecular Alterations in Bladder Tissue of Cftr^−^/^−^ Mice

**Figure 6:**
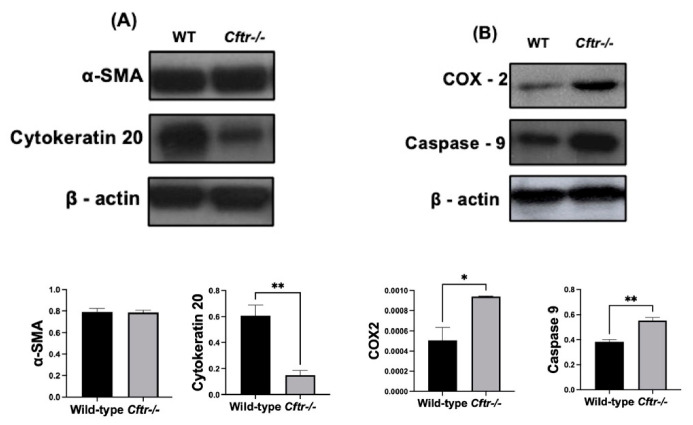
Molecular alterations in the bladder tissues of Cftr^−^/^−^ mice. (**A**) Representative Western blot images and corresponding quantitative analysis of α-smooth muscle actin (α-SMA) and cytokeratin 20 expression in bladder tissues of wild-type and Cftr^−^/α-SMA expression showed no significant difference between the groups, indicating preserved smooth muscle content, whereas cytokeratin 20 expression was significantly reduced in Cftr^−^/^−^ mice, suggesting alterations in the urothelial integrity. (**B**) Representative Western blot images and quantitative analysis of inflammatory and apoptotic markers. COX-2 expression was significantly increased in Cftr^−^/^−^ mice, indicating enhanced inflammatory signaling, whereas caspase-9 expression was significantly elevated, suggesting the activation of apoptotic pathways. β-actin was used as the loading control for normalization. Protein expression levels were quantified by densitometry using ImageJ software and were expressed relative to β-actin. Data are presented as mean ± SEM. Statistical significance was determined using unpaired Student’s *t*-test (* *p* < 0.05, ** *p* < 0.01).

Western blot analysis was performed to evaluate the molecular changes in the bladder tissue (Figure 6A,B).

The expression levels of α-SMA did not differ significantly between the groups, indicating the preservation of smooth muscle content. In contrast, cytokeratin 20 expression was significantly reduced in Cftr^−^/^−^ mice (*p* < 0.01), suggesting altered urothelial integrity and differentiation. In addition, COX-2 expression was significantly increased (*p* < 0.05), indicating enhanced inflammatory signaling, whereas caspase-9 levels were elevated (*p* < 0.01), consistent with the activation of apoptotic pathways. These findings indicate that CFTR deficiency is associated with urothelial alterations and the activation of inflammatory and apoptotic signaling pathways in the bladder tissue.

## 3. Discussions

The present study demonstrated that CFTR deficiency is associated with measurable disturbances in bladder functional physiology, as reflected by abnormal cystometric profiles, altered ex vivo detrusor contractility, modified ionic responsiveness, and selective changes in circulating electrolyte composition. These findings extend the functional relevance of CFTR beyond its established roles in classical epithelial organs and provide direct evidence that its loss influences lower urinary tract physiology. Although CFTR has been extensively studied in the airway [4] and pancreatic epithelia, its contribution to bladder function remains poorly defined. Therefore, the present results address an important gap by identifying a bladder phenotype detectable at both the whole-organ voiding behavior and intrinsic smooth muscle function levels. More broadly, these findings support the concept that epithelial ion transport pathways contribute to functional homeostasis of the bladder [15,16].

A key observation of this study was the altered cystometric pattern in Cftr^−^/^−^ mice, characterized by increased pressure fluctuations and irregular voiding contractions. These changes indicate a disruption in normal storage–voiding coordination rather than a structural defect in the bladder wall [17]. Cystometric abnormalities are commonly associated with dysregulated sensory signaling, altered detrusor excitability, and impaired neural coordination of the micturition reflex [18]. Consistent with this interpretation, histological and immunofluorescence analyses revealed a preserved bladder architecture and smooth muscle organization in Cftr^−^/^−^ mice. Together, these findings indicate that the observed bladder dysfunction is primarily functional rather than structural, likely due to altered regulatory mechanisms governing ion transport and excitability.

Ex vivo organ bath experiments were performed to further assess intrinsic detrusor function. High potassium stimulation induces contraction through membrane depolarization and activation of voltage-dependent Ca^2+^ channels, which is a central mechanism in detrusor excitation–contraction coupling [19]. In the urinary bladder, L-type Ca^2+^ channels play a dominant role in mediating this response [17]. In the present study, bladder strips from Cftr^−^/^−^ mice exhibited significantly reduced contractile responses to high-K^+^ stimulation, indicating impaired depolarization-dependent contractility. Because this approach bypasses receptor-mediated pathways, our findings suggest that CFTR deficiency affects intrinsic electrophysiological properties of smooth muscle, potentially through altered membrane excitability or ion handling. These observations are consistent with those of previous studies, highlighting the role of ion transport mechanisms in regulating bladder smooth muscle function [20].

At first glance, the coexistence of increased intravesical pressure in vivo and reduced contractility ex vivo appears to be contradictory. However, these findings reflect different levels of bladder function. Cystometry captures the integrated function of the lower urinary tract, including contributions from detrusor activity, urothelial signaling, afferent pathways, and neural reflexes [21], whereas organ bath experiments isolate intrinsic smooth muscle responses [19]. Altered urothelial signaling or sensory input may drive unstable bladder activity in vivo, whereas intrinsic smooth muscle responsiveness to depolarization is reduced [22]. 

A potential mechanism underlying these alterations is CFTR-mediated ion transport. CFTR functions as a chloride channel and modulates other transport systems, including the epithelial sodium channel (ENaC), thereby maintaining the epithelial ionic balance [15,23]. The loss of CFTR disrupts this regulation, potentially leading to altered extracellular ionic gradients [24]. Because detrusor contraction depends on membrane depolarization and Ca^2+^ influx, changes in the sodium, potassium, and chloride gradients can directly influence the membrane potential and excitation–contraction coupling [20].

Systemic electrolyte analysis revealed reduced sodium and chloride levels and elevated potassium concentrations in Cftr^−^/^−^ mice. Although these changes do not establish causality, they align with the known role of CFTR in epithelial ion transport [15,25]. Importantly, the extracellular ionic composition is a key determinant of smooth muscle electrophysiology, as variations in ionic gradients modulate membrane potential and calcium entry [26,27]. Even modest shifts in ionic balance can alter the threshold for detrusor activation [17]. However, systemic electrolyte measurements may not fully reflect the local ionic microenvironment within the bladder tissue, and further studies are warranted to establish direct mechanistic links.

In addition to functional and ionic alterations, CFTR deficiency is associated with distinct molecular changes in the bladder tissue. The expression of α-SMA remained unchanged, consistent with the preserved smooth muscle structure and the absence of overt histological remodeling [28]. In contrast, cytokeratin 20 expression was significantly reduced, suggesting an impaired urothelial differentiation. As umbrella cells play a critical role in maintaining the bladder barrier and regulating sensory signaling, the disruption of urothelial maturation may compromise barrier integrity and signal transduction [29]. The urothelium also functions as an active sensory interface, releasing mediators such as ATP, acetylcholine, and nitric oxide in response to mechanical and chemical stimuli [30,31]. Therefore, impaired urothelial differentiation may contribute to abnormal afferent signaling and bladder instability.

Previous studies have shown that CFTR negatively regulates COX-2/PGE2-mediated inflammatory signaling, and its loss enhances pro-inflammatory responses. Elevated COX-2 expression is consistent with inflammatory activation, whereas increased caspase-9 expression suggests the engagement of intrinsic apoptotic pathways [32]. These molecular alterations support the functional findings and suggest that CFTR deficiency influences bladder physiology through combined effects on epithelial integrity, ion homeostasis, and downstream cellular signaling.

In summary, the findings of this study indicate that CFTR deficiency produces a coordinated bladder phenotype characterized by ion imbalance, altered smooth muscle responsiveness, urothelial dysfunction, and activation of inflammatory and apoptotic signaling pathways. CFTR-mediated regulation of chloride transport and ionic balance appears to play a central role in maintaining detrusor excitability and bladder function [15,21]. Therefore, disruption of this regulation may influence bladder activity through integrated effects on ion transport, epithelial function and cellular signaling.

## 4. Materials and Methods

### 4.1. Animals

All animal procedures were conducted in accordance with national and institutional guidelines for the care and use of laboratory animals and approved by the Institutional Animal Care and Use Committee (IACUC No. A11438; approval period: 1 January 2026–31 August 2026), and they are reported in accordance with the recommendations of ARRIVE 2.0. Efforts were made to minimize animal suffering and use the minimum number of animals necessary. CFTR knockout mice on a C57BL/6 background and Slc9a3 knockout mice on a Friend Virus B (FVB) background were obtained from The Jackson Laboratory (Bar Harbor, ME, USA). These strains were maintained in our animal facility and crossbred to generate CFTR ^(+/+)^, ^(+/−)^, and ^(−/−)^ genotypes, as well as Slc9a3 ^(+/+)^, ^(+/−)^, and ^(−/−)^ genotypes. Genotyping was performed using genomic DNA extracted from tail biopsies and confirmed using polymerase chain reaction (PCR) analysis. Only genotypically verified CFTR^+/+^ and CFTR^−/−^ mice were included in this study. Sixteen male mice were used for functional analysis and equally divided into wild-type (CFTR^+/+^) and CFTR^−/−^ groups (*n* = 8/group). Five animals from each group underwent in vivo cystometry, followed by whole-blood collection for blood gas and electrolyte analyses. After euthanasia, bladder tissues were harvested and allocated for histological and immunofluorescence analyses and ex vivo organ bath contractility experiments (*n* = 6 per group). In addition, bladder tissues from a subset of three animals per group were processed for Western blot analysis (*n* = 3 per group), as described in Section 4.6 of the manuscript. The sample size was established through power analysis and consideration of prior studies, ensuring adequate statistical power to detect significant effects while adhering to ethical standards. Given the limited sample size, these analyses were considered supportive and interpreted alongside functional and histological outcomes rather than as independently powered endpoints. Slc9a3 knockout mice were generated during colony breeding and maintained for future investigations; however, they were not included in the present analysis. All mice were housed in IVC polycarbonate cages (2–4 per cage) at 22 ± 2 °C and 50–60% relative humidity, on corncob bedding with nesting material as environmental enrichment, in the SPF facility of the Fu Jen Catholic University Laboratory Animal Center. Standard rodent chow and reverse-osmosis water were provided ad libitum.

### 4.2. Genotyping

Genotyping was performed to confirm the CFTR genotype of each animal. Genomic DNA was extracted from tail biopsy samples using a standard extraction protocol with a commercially available kit (Cat. No. 101-T; Viagen Biotech, Inc., Los Angeles, CA, USA) following the manufacturer’s instructions. Polymerase chain reaction (PCR) amplification was conducted using primers specific for wild-type and knockout alleles: forward primer F1 (5′-CATACAACATAGGACTAGCC-3′), reverse primer R1 (5′-CACTACTAGTCAGGCACTCT-3′), and reverse primer R2 (5′-CACTTGTGTAGCGCCAAGTG-3′). PCR products (Genomics, New Taipei City, Taiwan) were analyzed by agarose gel electrophoresis, and the genotypes were determined based on the expected band patterns.

### 4.3. Cystometric Measurements

Bladder function was assessed using cystometric analysis to evaluate the urodynamic parameters in CFTR-deficient and wild-type mice [32]. Adult mice (approximately 12 months old) were anesthetized with a mixture of Rompun (5 mg/kg) and Zoletil (20 mg/kg) prepared at a 1:1 ratio and administered at a dose of 1 mL/kg body weight. The abdominal region was shaved and disinfected, and a midline incision was made to expose the bladders. A polyethylene catheter (PE-50) with a collar was inserted into the bladder dome and secured with a 6-0 polypropylene purse-string suture (Prolene; Cornelia, GA, USA). The catheter was tunneled subcutaneously, exteriorized through the thoracic region, and connected to PE-90 tubing for pressure recording. After catheter implantation, the incision was closed, and the animals were allowed to recover for five days. For cystometric recordings, the catheter was connected to a pressure transducer and an infusion pump. Sterile saline was infused into the bladder at a constant rate of 10–20 μL/min, and the intravesical pressure was continuously recorded. After stabilization, multiple micturition cycles were recorded. Key parameters, including the peak voiding pressure, were extracted to evaluate the bladder filling and voiding dynamics. 

### 4.4. Histological Examination

Histological analysis was performed to determine whether functional alterations were associated with structural changes in the bladder tissue. After the functional experiments, the mice were euthanized with an overdose of pentobarbital sodium. The bladders were excised, rinsed with phosphate-buffered saline (PBS), and fixed in 10% neutral-buffered formalin for 24 h. The tissues were dehydrated using graded ethanol, cleared in xylene, and embedded in paraffin. Sections (5 μm) were prepared, dewaxed in xylene, and rehydrated with graded ethanol prior to staining. For general histological evaluation, sections were stained with hematoxylin and eosin (H&E) to assess the overall bladder architecture, including the urothelium, lamina propria, and detrusor smooth muscle layers [32]. To further evaluate smooth muscle distribution, immunofluorescence staining for α-SMA was performed on the sections. Sections were incubated in a blocking solution containing 10% goat serum, 2% bovine serum albumin, and 0.2% Triton X-100 for 1 h at room temperature, followed by overnight incubation with the primary antibody at 4 °C. After washing with PBS, the sections were incubated with Alexa Fluor-conjugated secondary antibody for 1 h at room temperature. The nuclei were counterstained with 4′,6-diamidino-2-phenylindole (DAPI). Images were acquired using a fluorescence microscope (Leica DM2000 LED; Leica Microsystems, Wetzlar, Germany), and quantitative histomorphometric analysis was performed using ImageJ (National Institutes of Health, Bethesda, MD, USA) to determine the proportion of the α-SMA-positive area relative to the total tissue area.

### 4.5. Bladder Contractility/Organ Bath Assay

To determine whether CFTR deficiency affects intrinsic detrusor smooth muscle function, ex vivo bladder strip contractility was assessed using an organ bath system (OBS). After euthanasia, the bladders were excised and placed in cold Krebs solution. The connective tissue was removed, and longitudinal detrusor muscle strips were prepared for further analysis. The strips were mounted in organ bath chambers containing Krebs–Henseleit solution maintained at 37 °C and continuously aerated with 95% O_2_ and 5% CO_2_.

After equilibration for 30–60 min, contractile responses were induced using a high-potassium Krebs solution to depolarize the smooth muscle cells and evaluate ion-dependent contractility independent of receptor-mediated signaling [20]. The contractile force was recorded using an isometric force transducer. Peak tension was measured and compared between CFTR knockout and wild-type mouse ilea. Additional ionic stimulation conditions were used to assess the responsiveness of the detrusor smooth muscle to changes in the ionic environment. Data are expressed as mean ± SEM.

### 4.6. Western Blot Analysis

Western blot analysis was performed as previously described [32] to assess the proteins associated with smooth muscle structure, urothelial integrity, inflammation, and apoptosis. Bladder tissues were collected from wild-type and Cftr^−^/^−^ mice immediately after euthanasia and frozen in liquid nitrogen. Tissues were homogenized in ice-cold RIPA buffer containing protease inhibitors, and the lysates were centrifuged at 12,000× *g* for 15 min at 4 °C. The supernatants were collected for analysis.

Protein concentrations were determined using the bicinchoninic acid (BCA) assay. Equal amounts of protein (10 µg per sample) were denatured and separated by SDS-PAGE, followed by transfer onto PVDF membranes. The membranes were blocked with 5% non-fat milk in TBST and incubated overnight at 4 °C with primary antibodies against α-SMA (cat. no. 14395-AP), cytokeratin 20 (cat. no. sc-271183), COX-2 (cat. no. TA313292), and caspase-9 (cat. no. ab202068). After washing, the membranes were incubated with horseradish peroxidase-conjugated secondary antibodies for 1 h at room temperature. Protein bands were visualized using enhanced chemiluminescence and imaged with a chemiluminescence detection system. β-actin was used as the loading control. Band intensities were quantified using the ImageJ software Version 1.54g (National Institutes of Health, Bethesda, MD, USA), and protein expression levels were normalized to β-actin. Each experiment included at least three independent biological replicates (*n* ≥ 3 per group).

### 4.7. Statistical Analysis

All data are presented as the mean ± SEM. Statistical analyses were performed using GraphPad Prism version 10.4.1 (Dotmatics, Boston, MA, USA). Comparisons between groups were conducted using an unpaired Student’s *t*-test, with statistical significance set at *p* < 0.05. For Western blot analysis, comparisons were performed using *n* = 3 biological replicates/group. All other comparisons were performed using *n* = 8/group.

## 5. Conclusions

This study demonstrated that CFTR deficiency disrupts bladder homeostasis through coordinated functional and molecular alterations. These include abnormal cystometric patterns, altered detrusor contractility, modified ionic responsiveness, selective electrolyte imbalance, impaired urothelial differentiation, and activation of inflammatory and apoptotic signaling pathways. The preservation of α-SMA expression and bladder morphology indicates that these changes are primarily functional and cellular rather than structural changes. Overall, these findings support the role of CFTR-mediated ion transport in maintaining bladder physiology and suggest that its deficiency may influence bladder function through interactions between epithelial integrity, ion homeostasis, and downstream signaling pathways.

## 6. Novelty and Limitations of the Study

This study offers a comprehensive and integrative evaluation of bladder function in CFTR deficiency by combining cystometric analysis, ex vivo detrusor contractility assessment, systemic electrolyte profiling and molecular characterization. A key novel finding of this study was the identification of a multilevel bladder phenotype in CFTR deficiency, in which functional abnormalities were consistently linked to selective molecular alterations, including impaired urothelial differentiation and activation of inflammatory and apoptotic signaling pathways. In addition, this study provides previously unrecognized evidence that CFTR-mediated ion transport is closely associated with detrusor smooth muscle excitability, supporting the concept that epithelial ion channel dysfunction can influence bladder physiology through electrophysiological mechanisms rather than through overt structural remodeling.

Nevertheless, this study is primarily associative because the precise cellular pathways linking CFTR deficiency to altered contractile behavior were not directly examined. The relative contributions of the urothelial, smooth muscle, and neural components were not independently dissected, and it remains unclear whether systemic electrolyte alterations accurately reflect the local ionic environment within the bladder. In addition, the use of murine models necessitates further validation to determine the translational relevance of these findings to human bladder physiology.

## Data Availability

The datasets generated and analyzed in this study are available from the corresponding author upon reasonable request.
